# Diffusion-Weighted Imaging with Color-Coded Images: Towards a Reduction in Reading Time While Keeping a Similar Accuracy

**DOI:** 10.1155/2016/9592721

**Published:** 2016-03-13

**Authors:** Felipe Campos Kitamura, Srhael de Medeiros Alves, Luis Antônio Tobaru Tibana, Nitamar Abdala

**Affiliations:** Departamento de Diagnóstico por Imagem da Escola Paulista de Medicina da UNIFESP, Rua Napoleão de Barros, 800 Vila Clementino, 04024-002 São Paulo, SP, Brazil

## Abstract

The aim of this study was to develop a diagnostic tool capable of providing diffusion and apparent diffusion coefficient (ADC) map information in a single color-coded image and to assess the performance of color-coded images compared with their corresponding diffusion and ADC map. The institutional review board approved this retrospective study, which sequentially enrolled 36 head MRI scans. Diffusion-weighted images (DWI) and ADC maps were compared to their corresponding color-coded images. Four raters had their interobserver agreement measured for both conventional (DWI) and color-coded images. Differences between conventional and color-coded images were also estimated for each of the 4 raters. Cohen's kappa and percent agreement were used. Also, paired-samples *t*-test was used to compare reading time for rater 1. Conventional and color-coded images had substantial or almost perfect agreement for all raters. Mean reading time of rater 1 was 47.4 seconds for DWI and 27.9 seconds for color-coded images (*P* = .00007). These findings are important because they support the role of color-coded images as being equivalent to that of the conventional DWI in terms of diagnostic capability. Reduction in reading time (which makes the reading easier) is also demonstrated for one rater in this study.

## 1. Introduction

Nowadays there are many types of diffusion magnetic resonance imaging techniques, ranging from the most common—mapping apparent diffusion coefficient (ADC)—to the most advanced, such as diffusion spectrum imaging and tractography [1].

Diffusion-weighted imaging was first created for brain imaging application, but its use in other anatomical sites has gained wide acceptance due to its undeniable diagnostic contribution [2]. It is well known that signal intensities from corresponding voxels in high “*b*”-value image and its ADC map may predict tissue diffusion properties [3].

The most common use of these sequences is to analyze diffusion images with high “*b*”-values and compare them with its corresponding ADC map, side by side. As a general rule, high signal intensities in diffusion (high “*b*”-value) with low signal in ADC map are related to restricted diffusion, while low signal intensities in diffusion (high “*b*”-value) with high signal in ADC map are related to facilitated diffusion. Blackout effect is characterized when low signal intensities are seen at the same voxel both in diffusion-weighted images and at the ADC map; on the other hand, T2 shine-through effect happens when high signal is present at the same voxel in diffusion-weighted images and at the ADC map.

Therefore, it is known that a thorough voxelwise analysis of signal intensities in both images, side by side, is necessary to define tissue diffusion properties and aid radiologists in clinical practice. Even though comparing two images side by side does not take too long in the daily clinical practice, the diffusion sequences reading time could be reduced by combining the information of diffusion-weighted images and ADC map in a single image. Another advantage of combining both information items in a single image is related to the difficulty in analyzing the exact corresponding voxels when reading diffusion-weighted images and ADC map side by side. This is a major problem particularly when examining heterogeneous lesions. Reading a single image can make this task simpler.

The purposes of this study were (I) to develop a diagnostic tool capable of providing diffusion and ADC map information in a single color-coded image and (II) to assess the performance of color-coded images compared to their corresponding diffusion and ADC map.

## 2. Materials and Methods

### 2.1. Patients

The study was conducted in a tertiary teaching hospital with 712 beds. The institutional review board approved this retrospective study and waived the need for informed consent. Patients met the inclusion criteria if they were more than 18 years old and had been scanned with 1.5T MRI scanner. Patients were excluded if they lacked diffusion-weighted images with ADC maps or if these images had been made nondiagnostic by artifacts. The hospital's database was used to identify the list of patients who underwent head MRI between August 2015 and September 2015, sequentially enrolled, until sample size was reached.

### 2.2. Head MRI

All patients were imaged with the institution's protocol for head MRI, which included a diffusion-weighted sequence acquired in the axial plane, with three “*b*”-values (0, 500, and 1000). Two ADC maps were calculated: one of them was based on *b*0, *b*500, and *b*1000 while the other was based only on *b*500 and *b*1000. The MR scanner used was MAGNETOM Sonata 1.5T (Siemens Healthcare, Erlangen, Germany) with 8-channel head coil; matrix size of 128 × 128 pixels; FOV: 230 × 230 mm; slice thickness of 5.0 mm; and gradient strength of 40 mT/m.

### 2.3. Color-Coded Images

An algorithm was implemented in MATLAB® (MathWorks®, Natick, Massachusetts) to analyze the signal intensities from both diffusion-weighted images and ADC map of a given head MRI scan in order to generate a novel image that assigns a default color for each of the four main possibilities (restriction, facilitation, T2 shine-through, and blackout).

Diffusion sequences and corresponding ADC map were loaded into two three-dimensional matrices with the same size (128 × 128 × 20), one containing *b*1000 diffusion images (from now on referred to as diffusion) and the other containing the ADC map. Signal intensities of diffusion images were normalized for each axial slice and corrected through plane to account for signal inhomogeneities. In-plane signal inhomogeneities were not sufficiently intense in our images to cause artifacts in the color maps. A more robust correction may be needed to apply this technique to other scanners with different coil sensitivity profiles.

Initially, half-maximum values from normalized diffusion and ADC map in each slice were used as the “zero-effect” point, meaning that there is no restriction, facilitation, T2 shine-through, or blackout.

The following colors were chosen for each situation:

- Blue: restricted diffusion.
- Yellow: facilitated diffusion.
- White: blackout.
- Green: T2 shine-through.

These colors were chosen because they yielded good contrast, although any combination of colors could be used.

The Red, Green, and Blue (RGB) color system was used to represent color data, ranging from 0 to 1. In each slice, each voxel is first classified regarding its ADC value Then it is classified regarding its normalized *b*1000 signal intensity. Cut-offs were optimized empirically and became different than the initially mentioned “half-maximum values.” See Table 1 for details.

**Figure 1:**
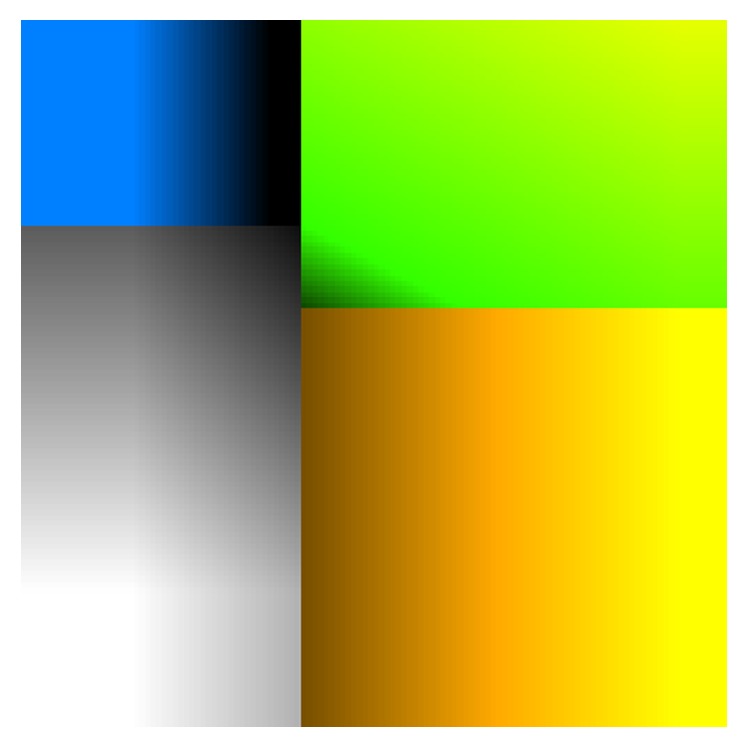
Resulting color map for a range of normalized *b*1000 signal intensities and ADC values. Normalized *b*1000 signal intensities range from 0 (lower part) to 1 (upper part). ADC values range from 0 (left) to 300 × 10^−5^ mm^2^/s (right).

After classification, a color is attributed to each voxel depending on the value of normalized *b*1000 (nDiff) and the normalized ADC (nADC). The formulas were empirically designed taking into account a simple idea:

(i) For restricted diffusion, as the ADC decreases below 120 × 10^−5^ mm^2^/s, the color of the voxel changes from black to blue. This produces a horizontal color gradient in the upper left corner of the color map in Figure 1. The formulas for restricted diffusion are
  (1) Red=0,Green=1−4nADC2,Blue=1−4nADC.
(ii) For facilitated diffusion, as the ADC increases above 120 × 10^−5^ mm^2^/s, the color of the voxel changes from dark yellow to light yellow. This produces a horizontal color gradient in the lower right corner of the color map in Figure 1. The formulas for facilitated diffusion are
  (2) Red=1.5nADC,Green=nADC,Blue=0.
(iii) For the T2 shine-through, both ADC and normalized *b*1000 were weighted to generate a color that changes from dark green to light green as the ADC and/or the *b*1000 signal increases. This produces a lower left to upper right diagonal color gradient in the upper right corner of the color map in Figure 1. The formulas for T2 shine-through are
  (3) Red=0.52nDiff+nADC−1.2,Green=2.52nDiff+nADC−1.2,Blue=0.
(iv) For the blackout, both ADC and normalized *b*1000 were weighted to generate a color that changes from dark gray to white as the ADC and/or the *b*1000 signal decreases. This produces a lower left to upper right diagonal color gradient in the lower left corner of the color map in Figure 1. The formulas for blackout are
  (4) Red=1−nDiff−nADC,Green=1−nDiff−nADC,Blue=1−nDiff−nADC.

The resulting matrix is multidimensional, accounting for 20 slices, 128 × 128 pixels, and 3 colors each (3 × 20 × 128 × 128).

A DICOM file was created for each resulting matrix, to facilitate reading of the resulting images by the radiologists.

Data manipulation, such as windowing the original data, or setting a different “zero-effect” point was necessary prior to the above mentioned algorithm, in order to accomplish better image contrast. Until now, this windowing process is done in a semiautomatic way.

### 2.4. Measurement of Interrater Agreement and Differences between Conventional and Color-Coded Images

Conventional and color-coded images were reviewed individually by 2 pairs of raters blinded to clinical data (pair A: raters 1 and 2: radiologists attending a neuroradiology fellowship; pair B: raters 3 and 4: first-year radiology residents). Interrater agreement was evaluated between each combination of raters (total of 6 combinations). Differences between conventional and color-coded images were assessed for each of the 4 raters. To avoid a learning effect, rating was done with conventional images first and a week later with the color-coded images. Scans were categorized into normal or abnormal, regarding the existence of an unexpected effect in a given location. Effect type (restriction, facilitation, blackout, and T2 effect) and location were also assessed to look for false-positive agreements.

### 2.5. Statistical Analysis

Cohen's kappa (*κ*) value and overall percent agreement (OPA) were used to quantify interrater agreement for both conventional and color-coded images. Also, positive percent agreement (PPA) and negative percent agreement (NPA) were used to compare the differences between conventional and color-coded images for all raters [4]. Bonferroni correction was applied to address the multiple comparisons problem. A two-tailed *P* value less than .05 was considered to be indicating a significant difference (before correction for multiple comparisons). After correcting for multiple comparisons, the cut-off became *P* < .0083. Sample size was estimated to guarantee 90% statistical power [5]. Rater 1 had his reading time computed and compared with paired-samples *t*-test.

## 3. Results and Discussion

### 3.1. Patients

Fifty-one patients who underwent head MRI between August 2015 and September 2015 were sequentially enrolled. After applying inclusion and exclusion criteria, there were 36 eligible scans, which was the sample size necessary to guarantee 90% statistical power.

### 3.2. Color-Coded Images

The algorithm generates a new color-coded image in which, by convention, restriction is shown in blue, facilitation is shown in yellow, T2 shine-through is shown in green, and blackout effect is shown in white.

**Figure 2:**
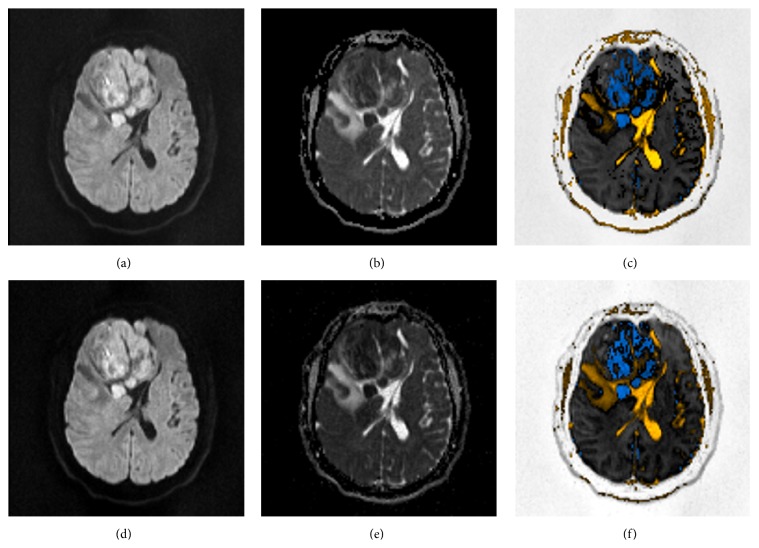
From (a) to (c): (a) high “*b*”-value diffusion (*b*1000). (b) Corresponding ADC map, calculated with *b*0, *b*500, and *b*1000. (c) Postprocessed image. Note that, for the window chosen, the tumor shows areas with different degrees of restricted diffusion (blue). Also, tumor heterogeneity can be better depicted, since corresponding voxels are matched exactly. Liquor appears as bright yellow. Vasogenic oedema near the tumor appears as dark yellow. From (d) to (f): (d) high “*b*”-value diffusion (*b*1000). (e) Corresponding ADC map, calculated with *b*500 and *b*1000, without *b*0. (f) Postprocessed image. All images were shown in the same window.

**Figure 3:**
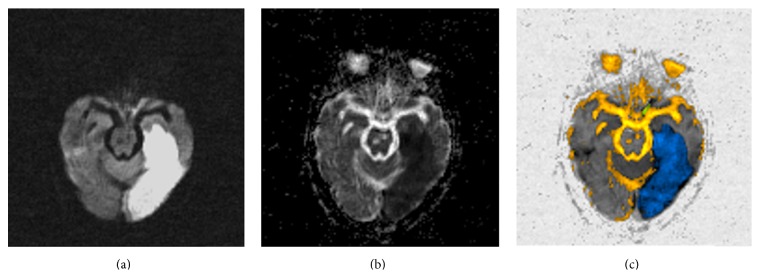
(a) High “*b*”-value diffusion (*b*1000). (b) Corresponding ADC map. (c) Postprocessed image. This is an acute left posterior cerebral artery stroke (blue). Note chronic lacunar infarcts in the pons (yellow).

**Figure 4:**
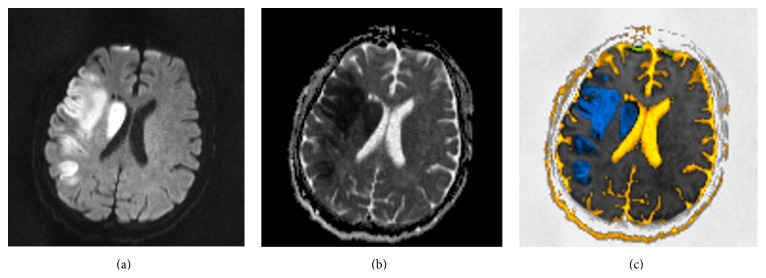
(a) High “*b*”-value diffusion (*b*1000). (b) Corresponding ADC map. (c) Postprocessed image. This is an acute right middle cerebral artery stroke (blue).

Examples are given in Figures 2, 3, and 4, demonstrating *b*1000 diffusion image, ADC map, and the resulting color-coded image. In general, ADC maps calculated with *b*500 and *b*1000 (without *b*0) were no different than their respective ADC maps calculated with *b*0, *b*500, and *b*1000, particularly in strokes and in normal scans. This was also true for the corresponding color-coded images. Tumors showed slightly lower ADC values when calculated only with *b*500 and *b*1000, as depicted in Figure 2.

As expected, any factor that deteriorates the diffusion images will also affect the color-coded images. Several factors may contribute to the generation of artifacts in DWI: limitations from the gradient system hardware (gradient amplitude, slew rate, nonlinearity, and instability) may cause image distortion and widely distributed ghost artifacts; Eddy currents from diffusion sensitizing gradients may cause geometric distortion (contraction, dilation, shift, and shear); Eddy currents from echo-planar imaging (EPI) may cause “N/2” ghost; the low bandwidth of echo-planar imaging (EPI) in the phase-encoding direction may cause significant displacement of fat in this direction, due to chemical-shift; also, the low bandwidth of EPI in the phase-encoding direction causes severe shape distortion in this direction, because, even in a well-shimmed magnet, the human head will magnetize unevenly, specially in tissue interfaces with very different magnetic susceptibilities, like bone and air [6].

**Figure 5:**
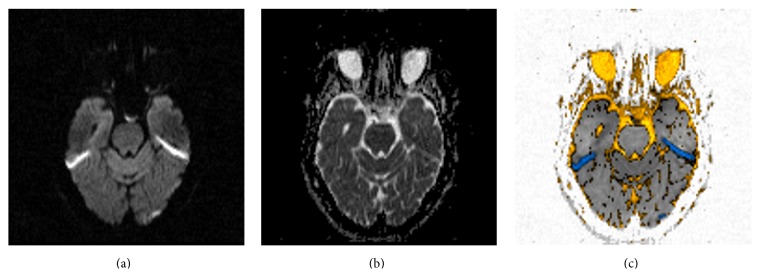
(a) High “*b*”-value diffusion (*b*1000). (b) Corresponding ADC map. (c) Postprocessed image. This is an example of spatial misregistering due to susceptibility artifacts induced by the temporal bones.

Considerable progress has been made in the last 30 years to develop high-performance gradient coils available today, capable of providing linear and stable gradients of the utmost intensity (at least 40 mT/m). Adding preemphasis to the gradient shape and using “self-shielded” gradient coils helped to manage Eddy current and its artifacts. The fat misregistration due to chemical-shift can be eliminated in a straightforward manner by applying a fat-saturation pulse prior to imaging. Besides, using lower *B*_0_ field strength (1.5T instead of 3T) can aid in reducing field inhomogeneities. All these techniques were applied to our images. However, spatial misregistering in the phase-encoding direction due to local field inhomogeneities is still difficult to overcome [6]. An example of this last artifact is shown in Figure 5.

### 3.3. Differences between Conventional and Color-Coded Images

*Pair A*. For rater 1, conventional and color-coded images had almost perfect agreement (OPA = 91.7%; PPA = 100.0; NPA = 78.6; *κ* = .818; and *P* = .000001). For rater 2, conventional and color-coded images had substantial agreement (OPA = 86.1%; PPA = 82.6%; NPA = 92.3%; *κ* = .713; and *P* = .000014).

*Pair B*. For rater 3, conventional and color-coded images had substantial agreement (OPA = 86.1%; PPA = 89.3%; NPA = 75.0%; *κ* = .615; and *P* = .000213). For rater 4, conventional and color-coded images had almost perfect agreement (OPA = 91.7%; PPA = 100.0%; NPA = 83.3%; *κ* = .833; and *P* < .000001).

After reviewing the 16 cases in which the raters disagreed, a fifth rater considered the color-coded images to be the right ones in 8 of these cases.

There is substantial or almost perfect agreement between the reading of conventional and color-coded images for all raters. The agreement index (*κ*) does not seem to correlate with the rater's experience, since the greatest and the lowest *κ* were found in the least experienced pair of raters (B). These findings are important because they support color-coded images as being equivalent to conventional DWI in terms of diagnostic capability. Reduction in reading time is also demonstrated for one rater in this study, which supports the idea of reading color-coded images being a simpler task when compared to the reading of conventional DWI images.

### 3.4. Interrater Agreement

In pair A (raters 1 and 2), interrater agreement (*κ*) was statistically significant with conventional images (OPA = 75.0%; *κ* = .467; and *P* = .005) and with color-coded images (OPA = 80.6%; *κ* = .594; and *P* = .000198).

In pair B (raters 3 and 4), interrater agreement (*κ*) was not significant with conventional images (OPA = 66.6%; *κ* = .333; and *P* = .016) but significant with color-coded images (OPA = 83.3%; *κ* = .636; and *P* = .000042).

For raters 1 and 3, interrater agreement (*κ*) was significant with conventional images (OPA = 77.7%; *κ* = .493; and *P* = .001) and with color-coded images (OPA = 94.4%; *κ* = .862; and *P* < .000001).

For raters 1 and 4, inter-rater agreement (*κ*) was significant with conventional images (OPA = 83.3%; *κ* = .667; *P* = .000041) and with color-coded images (OPA = 88.9%; *κ* = .762; *P* = .000002).

For raters 2 and 3, interrater agreement (*κ*) was not significant with conventional images (OPA = 75.0%; *κ* = .409; and *P* = .009) but significant with color-coded images (OPA = 75.0%; *κ* = .471; and *P* = .002).

For raters 2 and 4, interrater agreement (*κ*) was significant with conventional images (OPA = 75.0%; *κ* = .500; and *P* = .002) and with color-coded images (OPA = 86.1%; *κ* = .717; and *P* = .000016).

Interrater agreement is significant for all the 6 pairs of raters when analyzing color-coded images, but not when analyzing conventional images, as seen between rater 3 and raters 2 and 4. Also, interrater agreement index (*κ*) was higher for color-coded images than for conventional DWI for all the 6 pairs of raters. These findings support the hypothesis that color-coded images would be more reproducible than conventional DWI, especially for less experienced radiologists.

Mean reading time of rater 1 was 47.4 seconds for conventional DWI and 27.9 seconds for color-coded images (*P* = .00007).

Another important aspect of this work is the potential to avoid misinterpretation, either by inattention or by those who are not used to reading conventional DWI, as was observed with the 8 cases in which color-coded images were deemed as correct.

Previous authors have demonstrated that using color-coded images increased interrater agreement of FLAIR images and the correlation between time from stroke onset and FLAIR signal. It also yielded higher specificity and positive predictive value for the identification of patients with ischemic stroke within 4.5 h of symptoms onset [7], thus, providing evidence that color-coding images may improve the accuracy of radiologists in reading magnetic resonance scans.

However, the authors in that study applied a different approach for the color-coding FLAIR images method: they applied a color map to a single sequence, which only adds subjective effects in the reading process. The information contained in that color-coded image is essentially the same as that of the conventional one. Our method is different because it combines two source images (high “*b*”-value DWI and ADC map) to assemble a new one, providing an image with higher information content.

Our study had some limitations. First, one of the raters had already seen three cases among our sample, making it more susceptible to remember clinical and/or image details of the cases, which would make it easier to detect abnormalities in that study. Second, all scans were acquired with only one MRI scanner, which limits extrapolation to scanners from other companies. Third, reading time was assessed only for one observer, limiting conclusions about time reduction.

Our paper, while important, demonstrates the need for additional, multiple reader and, ideally, “perfect-referenced” study.

## 4. Conclusion

Interpretation of color-coded images seems to be equivalent to or even better than the interpretation of conventional DWI/ADC in terms of reading time and reproducibility.

## Figures and Tables

**Table 1:** Voxel classification depending on its ADC and on its normalized *b*1000 signal intensity.

ADC (10^−5^ mm^2^/s)	Normalized *b*1000 signal	Effect
≥120	≥0.6	T2 shine-through
	<0.6	Facilitated diffusion

<120	≥0.64	Restricted diffusion
	<0.64	Blackout
